# Online Assessment of Morphological Awareness in Grades 2–4: Its Development and Relation to Reading Comprehension

**DOI:** 10.3390/jintelligence10030047

**Published:** 2022-07-25

**Authors:** Szilvia Varga, Attila Pásztor, János Stekács

**Affiliations:** 1 Language Teaching and Examination Centre, John von Neumann University, 6000 Kecskemét, Hungary; 2 MTA—SZTE Metacognition Research Group, 6722 Szeged, Hungary; steklacs.janos@pte.hu; 3 Institute of Education, University of Szeged, 6722 Szeged, Hungary; attila.pasztor@edu.u-szeged.hu; 4 MTA—SZTE Digital Learning Technologies Research Group, 6722 Szeged, Hungary; 5 MTA—SZTE Research Group on the Development of Competencies, 6722 Szeged, Hungary; 6 Institute of Education, University of Pécs, 7624 Pécs, Hungary

**Keywords:** morphological awareness, reading, linguistic intelligence, online assessment

## Abstract

The aims of the study are to construct an online instrument to assess different aspects of morphological awareness and to examine its development and its relation to reading comprehension in grades 2–4 in Hungarian children. Altogether, 4134 students were tested. The online test evaluated inflectional, derivational, and compound morphological skills with five subtests. The instrument proved to be reliable. CFA examinations revealed that the five subtests were empirically distinguishable dimensions. Inflectional, derivational, and compound morphology as the three main dimensions of morphological awareness were also empirically supported by our data. Morphological awareness skills improved significantly and developed in parallel with reading skills throughout grades 2–4. The increase in the development of morphological awareness from grade 2 to grade 3 tends to be faster than the growth between grade 3 and 4. Positive moderate correlations were found between morphological skills and reading comprehension and the relationships seem to be stable throughout the three grades. The most significant predictor of reading comprehension is the Affix Identification for Nonwords subtest. Our study showed that morphological awareness could be assessed efficiently through online media and drew attention to the importance of morphological awareness in the development of reading comprehension and linguistic intelligence.

## 1. Introduction

Intelligence is a multiple construct; there is still an ongoing discussion about its definition, underlying mechanisms, and structure (e.g., Kovacs and Conway 2019). However, there is a consensus that language skills play an important role in general intelligence. For instance, language skills such as grammatical sensitivity, language development, and reading and writing are all part of the Cattell–Horn–Carroll’s Theory of Cognitive Abilities (Flanagan and Dixon 2014; McGrew 2005). Furthermore, linguistic intelligence is one of the key factors in Gardner’s Theory of Multiple Intelligences (Gardner 2000). Linguistic intelligence is the ability to communicate through the language, which involves reading, writing, listening, and speaking. It facilitates the improvement of cognitive skills, which can assist in arranging thoughts as well as sharpen analytical skills (Gardner 2000; Kornhaber 2019). Furthermore, a great number of research studies found that both language skills and reading skills relate to the construct of intelligence (Baghaei and Tabatabaee 2015; Federmeier et al. 2020; Mirsaeedghazi 2021; Schipolowski et al. 2014). Morphological awareness refers to the skill of reflecting upon and manipulating morphemes and employing word formation rules in one’s language (Kuo and Anderson 2006). Morphological awareness is predictive of future reading; therefore, its diagnostic assessment promotes identifying children with reading difficulties (Enderby et al. 2021; Levesque et al. 2021). It has an impact not only on reading skills but on lexical acquisition, too (Rajab 2020). Morphological awareness enables students to guess the meaning of unfamiliar words since children learn to identify the stem and the affixes. By acquiring morphological skills, students gain the knowledge about the syntactic functions of morphemes (Carlisle 2010).

Morphological awareness heavily depends on the underlying mechanism of general intelligence, such as on different aspects of working memory (Baddeley et al. 2021; Sternberg 2022). For example, students have to identify the different sounds in the stem and the affixes by relying on the processes of phonological awareness and phonological memory. The development of morphological awareness also depends on the capacity of working memory and on the processes of executive functions such as manipulating and integrating information and monitoring the task-solving behavior. Thus, morphological awareness is a construct belonging to language awareness which can be associated with different processes of general intelligence and linguistic intelligence. Assessing different aspects of morphological awareness and examining its development may extend our knowledge on the understanding of general cognitive processes as well.

The objective of the large sample assessment in the current study was to tap into the structure of morphological awareness and examine its development in grades 2–4. Also, the large sample survey gave an additional major insight into how the relationship between morphological awareness and reading comprehension changes in grades 2–4.

### 1.1. Definition and Development of Morphological Awareness

There is no consensus about the definition of morphological awareness (Apel 2014). Most definitions use the term ability or skill when they refer to morphological awareness (Apel 2014; Carlisle 2000; Deacon et al. 2014; Nagy et al. 2014). We based our investigations on the definition proposed by Kuo and Anderson (2006). They define it as the ability to reflect upon and manipulate morphemes and employ word formation rules in one’s language (Kuo and Anderson 2006). Three dimensions of morphological awareness have been identified: inflectional, derivational, and compound morphology (Kuo and Anderson 2006). Inflectional morphology includes changes made in existing words; inflections express grammatical categories such as tense, aspect, gender, case, or number. Derivational morphology deals with the creation of new words; they can be formed by a number of formal means such as affixation, internal modification of different words, subtraction, and conversion. Compounding is also a word formation process grounded in a combination of lexical elements (e.g., words, roots).

Morphological awareness allows decoding multisyllable, novel, and nonsense words by analogy (Gabig and Zaretsky 2013; Kuo and Anderson 2006). Morphological awareness becomes important, in full-alphabetic and consolidated-alphabetic phases of decoding, when the sequence of letters in a word becomes salient. A student groups common patterns of letters and sounds as units. They decode many words by sight (Ehri 2005; Levesque et al. 2021). Morphological decomposition is essential in understanding the systematic relationships among the surface forms of the words and their meaning (Verhoeven and Perfetti 2017).

The different dimensions of morphological awareness (inflectional, derivational, and compound morphology) show gradual development throughout primary grades. Children acquire inflectional morphology earlier than compound and derivational morphology. The development of morphological awareness involves three major aspects: relational, syntactic, and distributional knowledge, which appear in the acquisition of inflectional, derivational, and compound morphology (Apel 2014). The three subskills of morphological awareness show the greatest growth during the first three or four grades. However, derivation even shows significant growth after grade 4 (Berninger et al. 2010).

Grade-specific guidelines for the development of morphological knowledge and awareness are described in Common Core State Standards for English Language and Arts (CCSSI 2011). These standards refer to English, which is a deep orthography. However, there is a lack of research on the development of morphological awareness in shallow orthographies. The development of morphological awareness has received more research attention in deep orthographies than in shallow orthographies. In shallow orthographies, such as in Finnish, Turkish, or Hungarian, there is a consistent relationship between graphemes and phonemes. In deep orthographies such as English, the relationship between phonemes and graphemes is not transparent; it is sometimes difficult to predict the pronunciation from spelling of a word.

In both shallow and deep orthographies, explicit knowledge of morphological awareness starts developing in grade 1 when children learn how to decode (read) words into sounds and encode (write) words into visual symbols (Carlisle 2000; Manolitsis et al. 2019). They start recognizing the orthographic patterns which represent, for example, past tense, plurality, and possession. They are expected to acquire the relational knowledge among common words with various grammatical inflections which mark tense, number, and grammatical function. Additionally, the awareness of lexical morphology starts to emerge, and children start decomposing and blending compounds (Gabig and Zaretsky 2013). In grade 2, children are expected to acquire the basics of the derivational morphology. They learn the most common prefixes and suffixes and start decomposing complex words. In grade 3, children can identify the meaning of the majority of common prefixes and suffixes and analyze the suffixes and prefixes within a novel word. In grades 4-6, relational knowledge and syntactic knowledge of Greek and Latin origin morphemes start evolving (Gabig and Zaretsky 2013).

### 1.2. Acquisition of Morphology in Shallow Orthographies

Learning to read has some common features which can be applied for all the alphabetic languages; however, some differences were found in the case of languages with different writing systems. This view is supported by the universal grammar theory, which claims that the basic processes of comprehension are similar for all human languages (Chomsky et al. 2019; Dąbrowska 2015). Although the basic processes of reading acquisition in languages with different writing systems are the same, the different phases of reading development can be different (Verhoeven and Perfetti 2017). For example, Finnish, Turkish, and Hungarian children learn to read compound words and even nonwords quite early, and can read many types of word endings with different suffixes. Finnish children can read fluently after one month of reading instruction (Holopainen 2002). It seems probable that morphological processing plays an important role in reading development after the initial skill of phonological awareness is acquired (Aro 2017). In Turkish literacy acquisition, morphology plays an important role (Durgunoğlu 2017). Haddad et al. (2018) showed that children can benefit greatly from morphological segmentation in a shallow orthography. These results are in line with previous studies demonstrating the significance of morphological segmentation even in shallow orthographies such as Italian and French (Casalis et al. 2015).

In Hungary, there is a lack of research on morphological awareness. There are some basic requirements related to grammatical skills. The Hungarian National Core Curriculum (HNCC) (2020) suggests fostering grammatical skills in a playful way rather than repeating grammatical rules. The Standardized Framework Curriculum for Grades 1–4 (SFC 2020) mentions the importance of developing metalinguistic awareness skills. In grade 2, children start being familiar with term-stems and affixes. They acquire inflectional morphology and recognition of basic suffixes. They are supposed to learn morpheme segmentation, and they are introduced to homonyms. The curriculum includes the recognition of verbal inflections, for example, the inflections for present, past, and future tenses. They become familiar with decomposing nouns and adjectives. Nominal inflections such as plurals are identified. They learn derivation in grade-level texts. In grade 4, compound morphology is mastered. Children must acquire syntactic functions of morphemes, and they must recognize and decompose different parts of speech (nouns, verbs, adjectives). Numerals and postpositions are recognized as well.

In shallow orthographies, children can usually spell printed words without analyzing the morphological structure of the word. However, morphological knowledge becomes important when children must comprehend more-complex structures, and have to understand polymorphemic words (Babayigit 2009). It has been argued that morphological awareness works similarly in deep and shallow orthographies; thus, more empirical research would be needed to explore how morphology takes part in this process (Verhoeven and Perfetti 2017).

### 1.3. Contribution of Morphological Awareness to Reading Comprehension

The PIRLS Framework for Assessing Reading Achievements provides international standards for reading comprehension performances in grade 4. PIRLS defines reading comprehension as follows: “Reading literacy is the ability to understand and use those written language forms required by society and/or valued by the individual. Readers can construct meaning from texts in a variety of forms. They read to learn, to participate in communities of readers in school and everyday life, and for enjoyment” (Mullis and Martin 2021, p. 6). The PIRLS survey focuses on retrieving explicitly stated information; it also evaluates the ability to make straightforward inferences, interpret and integrate ideas and information, and evaluate and critique content and textual elements.

A number of research studies pointed out the significance of morphological awareness in reading comprehension (Casalis et al. 2011; Deacon et al. 2014; James et al. 2021; Levesque et al. 2021; Liu et al. 2017; Singson et al. 2000). School-age children infer meanings of new words based on word structure (Wolter and Green 2013). The hypothesis that morphological awareness can influence reading comprehension both directly and indirectly has been supported by current findings (Levesque et al. 2017). Morphological knowledge influences word identification, spelling, and word reading (Enderby et al. 2021), which affects reading comprehension. It has an impact on cognitive and linguistic skills which support reading comprehension (Liu et al. 2017). Children’s morphological awareness shows growth throughout primary school years, and it becomes an increasingly strong factor of reading comprehension performances and academic achievements (Levesque et al. 2017; James et al. 2021; Meaux et al. 2020). Morphological instruction primarily supports reading comprehension possibly in a bidirectional way (Levesque et al. 2021; Manolitsis et al. 2019). However, there might be a difference between the strength of this relationship in different orthographies (Manolitsis et al. 2019). Studies suppose that children who study reading in shallow orthographies develop coding skills earlier than in deep orthographies (Borleffs et al. 2019). In shallow orthographies, morphological skills are not necessary for decoding; however, morpheme segmentation skills become important for reading pseudowords (Haddad et al. 2018). Mousikou et al. (2020) examined whether morphological processing in reading is influenced by the orthographic consistency of a language or its morphological complexity. They found that the orthographic consistency of a language, rather than its morphological complexity, determines the extent to which morphology is used during reading (Mousikou et al. 2020).

### 1.4. Assessment of Morphological Awareness

Diagnostic tests for assessing the three dimensions of morphological awareness (inflectional, compound, and derivational morphology) at school are applied to provide detailed information about the construct (Apel et al. 2013; Carlisle 2000; Deacon et al. 2014; Kuo and Anderson 2006; Levesque et al. 2017; Rastle 2019). Morphological awareness has been assessed orally, in a written form, or with a mixed method, i.e., a combination of oral and paper-based methods. Apel et al. (2013) list several typical tasks for assessing morphological awareness. Judgment and production tasks are implemented to test the ability both on real- and nonwords. In judgment tasks, children have to identify morphemes or semantic or syntactic relationships among different words. Affix identification and suffix choice tasks can be presented orally or in writing within a multiple-choice pattern or by answering questions or completing sentences (Kuo and Anderson 2006; Apel 2014). In addition to real-words tasks, nonwords tasks have also been applied. In nonwords tasks, children are not able to rely on the meaning of the words (Apel 2014). These tasks proved to be especially effective for the assessment of word reading skills, and they are closely related to reading comprehension performances (Guldenoglu et al. 2012). There are several types of production tasks. In these tasks, children are expected to manipulate morphemes e.g., complete words or sentences (cloze tasks) or explain the meanings of morphemes (dynamic assessment task). Blending and segmenting tasks assess how children can form new words by combining morphemes or how they can decompose words into the constituent morphemes (Apel 2014). Analogy tasks require children to use analogies given by the examples (Apel 2014; Kirby et al. 2012). These assessments use face-to-face methods and children are tested individually.

The administration of these paper-based or oral tests is time consuming and because of the delayed feedback, they cannot be applied regularly for diagnostic aims in school settings. The advantages of computer-based testing may provide solutions for these problems. Additionally, there are situations when face-to face methods do not work, such as during the COVID-19 pandemic. The new information—communication technologies have greatly contributed to the improvement of pedagogical assessment. Technology offers new assessment methods that change education assessment from authoring to the automatic generation and storage of items through the delivery methods (Csapó and Molnár 2019). Online assessment is relatively cost-effective since it reduces the costs of printing (Dhawan 2020). Online assessment facilitates quick and clear reports on the students’ results and progress. It makes it easier to give useful feedback to the students who are making progress in skills or subskills, and helps identify which areas of learning require attention (Dhawan 2020; Shute and Rahimi 2017; Snyder et al. 2005). Thus, online assessment tools could greatly increase the number of children who could receive objective feedback on their morphological skills, and call attention to the educators about the importance of morphological skills. However, there are no online instruments for measuring morphological awareness for students in grades 2–4. Therefore, creating a valid online assessment tool seemed to be a fruitful endeavor.

### 1.5. Objectives of the Present Study

The aims of the study are to construct an online instrument to assess different aspects of morphological awareness and to examine its development and its relation to reading comprehension in grades 2–4 in Hungarian children. The Hungarian language has a shallow orthography; thus, the study could provide further empirical findings about the nature and development of morphological awareness. Our research questions are related to the instrument, the structure, the dimensions of operations of morphological awareness, and its development. Also, the relationship between morphological awareness and reading comprehension in grades 2–4 is further explored. More specifically, we aim to answer the following research questions:

1. What are the psychometric features of the test?
2. How do morphological awareness skills develop in grades 2–4?
3. What relationship does morphological awareness have with reading comprehension throughout grades 2–4?

In the case of the second research question, it can be assumed that there will be a difference in the levels of development of morphological awareness in the different grades (Carlisle 2000). The development of inflectional morphology precedes the development of the compound and derivation morphology. Therefore, better results might be expected in the affix identification subtests than in compound and derivation subtests. In the case of the third research question, we hypothesize that there will be moderate correlations between different aspects of morphological awareness and reading comprehension (Kirby et al. 2012). These correlations will increase throughout primary school years and will become an increasingly strong factor in reading comprehension performances (Levesque et al. 2017).

## 2. Materials and Methods

### 2.1. Participants

The sample of the study was drawn from second, third, and fourth grade students, altogether having 4134 children (2026 boys and 1877 girls). The number of students, their age, and their gender distribution in different grades are shown in Table 1. The sampling units were the school classes, and altogether, 256 classes from 94 Hungarian schools were examined (on average 16.1 students in one class, SD = 7.1). The sampling procedure was nationwide; schools voluntarily participated in the study from various regions of Hungary.

### 2.2. Instruments

The instrument focused on the evaluation of different subskills integrated into morphological awareness. It encompassed different aspects of morphological awareness assessing inflectional, derivational, and compound morphology through testing relational, syntactical, and distributional knowledge. The test covered a wide selection of subskills related to morphological awareness. Three aspects of the construct were measured with five subtests. Inflectional morphology was tested by affix identification with real- and nonwords; derivational morphology was tested with the derivation and segmentation subtests. Compound morphology was tested with the compound-words subtest. The online instrument contained 60 items and consisted of five subtests. It included 12 items in each subtest: identification of affixes for nonwords (Berko 1958; Apel 2014) and for real words (Kirby et al. 2012), compound words (Apel et al. 2013; Berko 1958; Carlisle 2000; Nippold and Sun 2008; Tyler and Nagy 1989), derivation (Carlisle 2000; Nippold and Sun 2008; Tyler and Nagy 1989), and a morpheme segmentation subtest (Apel et al. 2013; Carlisle 2000). Inflectional, compound, and derivational tasks were based on traditional instruments applied for the assessment of morphological awareness (Apel 2014; Carlisle 2000). Frequent words and common affixes were used in the test. In the nonwords tasks, basic inflectional suffixes were included.

The affix identification for nonwords subtest aimed to demonstrate how efficiently children could identify different morphemes when they could not rely on the meaning of the word. Children had to choose the correct word or sentence out of four alternatives. The sentences consisted of one or two nonwords. Real inflections were attached to the nonwords. This task included a number of language elements: children had to identify the correct noun and verb inflections. The inflections of the present and past tenses of verbs, singular and plural nouns, and objective and instrumental cases of nouns had to be identified, which are also regarded to be common inflections (Oravecz et al. 2014). The first three items were comprised of the identification of inflections for single and plural nouns. Children had to choose grammatically correct sentences including inflected nonwords from four alternatives (Berko 1958, Figure 1). In the affix identification for the real-words task, children had to put the words in the menu into two boxes: one box was for the root words and one for the affixed words.

In the compound-words subtest, the awareness of compound morphology was assessed. Children saw four words on the monitor, and they had to click on the compound word. Each item consisted of one real compound, a pseudo compound, and inflected or derived words (Figure 2). In the derivation task, students had to identify the correct derived word from four options (Apel et al. 2013; Berko 1958; Nippold and Sun 2008; Figure 2). In the derivation subtest, only productive derivational affixes were used. The traditional instruments (Carlisle 2000) use different types of production type tasks (sentence completion). Children have to complete the sentence with the correct derived word: farm (My father is a/an ........). Traditional testing uses a wide range of oral tasks, such as morphological analysis (Levesque et al. 2017) in which children have to explain the meaning of different morphemes. These tasks work in oral testing; however, they are not operable in online assessment.

The morphological segmentation subtest consisted of two parts: in the first part, children had to make a decision about how many parts the words had (1–4). Real affixes were attached to pseudowords; for example, zelenálok. The answers were given by clicking on the correct number (1, 2, 3, or 4). In the second morpheme-segmentation task, we applied real words similar to the one used by Carlisle (2000); this is called the “comes from task” (Figure 3). These relational tasks assessed the awareness of identification of multimorphemic words. The children had to identify whether the first word was formed from the second word or not. They answered by clicking on the word yes (*igen*) or no *(nem).* The subtest was challenging since the second word was orthographically similar to the first word; for example, *szellő*, *szel*; *körte*, *kör*. Morpheme segmentation was originally an oral task used by a number of researchers (Apel et al. 2013). In traditional instruments, in a “comes from task”, students have to make a decision about the semantic relation between two words. It is also called the “Does this word come from the other word?” task; for example: “Does mother come from moth?” (Kuo and Anderson 2006). A usual answer for this question is: yes or no. Another task involved in judgment of semantic accuracy is a sentence completion task. There are four options, and children have to complete the sentence: (“direct, directing, directed, directions) did you understand the _______?” (Apel et al. 2013; Nippold and Sun 2008). Table 2 shows the subtests of the online morphological awareness test. Trial items were also developed for all subtests in order to increase the validity of the test results.

The instrument also included a basic reading comprehension skills test in order to reveal the relationship between children’s morphological and reading skills. The test evaluated the basic level of reading comprehension (searching for keywords, skim reading, and scanning), and students needed to process information and facts explicitly stated in the text. Thus, according to the PIRLS definition, the test we designed assessed how children could retrieve explicitly stated information and make straightforward inferences (Mullis and Martin 2021). basic competence in literal reading skills is required by the curriculum in grade 2. The test included a short, simplified text with ten multiple-choice questions; students were expected to elicit explicit facts stated by the text. The text contained 161 words, and it was a description of an imaginary international festival for children. The vocabulary of the text contained common expressions and activities. On the top of the screen, children could read the text for each question which was displayed at the bottom of the screen. The instruction was the following: “Read the following text and answer the question by clicking on the correct answer”. Sample question: “How often is the festival organized? (a) every year (b) every six months (c) every month (d) every second year”. It was an important aim that students could complete both the morphological test and the reading-comprehension tasks in a 45 min lesson. Therefore, only a short reading test could be conducted within this time frame.

### 2.3. Procedures

**Figure 1:**
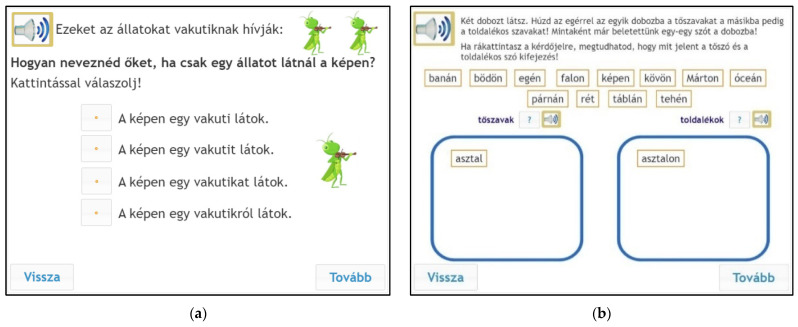
Sample items for affix identification. Left (**a**): nonwords, instruction: “These animals are vakutis. What do you say if there is only one animal in the picture? Answer by clicking”. Options: I can see vakuti in the picture/I can see a vakuti in the picture/I can see a vakutis in the picture/I can see about vakutis in the picture. Right (**b**): real words, instruction: “You can see two boxes on the screen. Drag the base words into one box and the inflected words into the other box. We have already put one word into each box as an example. If you click on the question mark, you can learn what base word means and what affixed word means”. Options: banana, pot, in the sky, on the wall, in the picture, on the stone, Martin, ocean, on the pillow, meadow, on the board, cow.

**Figure 2:**
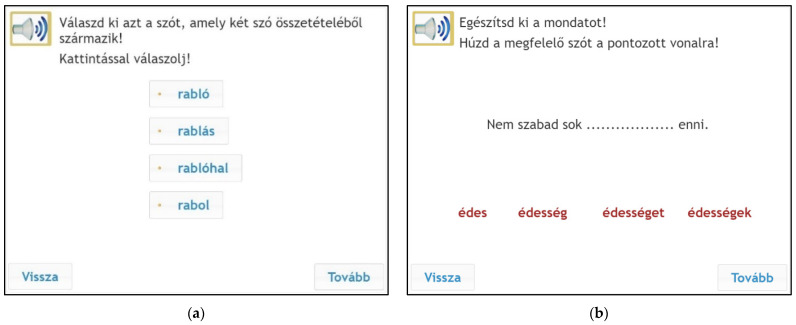
Sample items for compound words and derivation. Left (**a**): compound words, instruction: Choose the compound word which is made up of two words. Answer by clicking on the correct word. Options: robber, robber fish, robbery, rob. Right (**b**): derivation, instruction: “Complete the following sentence. Drag the appropriate word onto the dotted line”. Options: You must not eat a lot of sweet/sweetness/sweets/sweetnesses.

**Figure 3:**
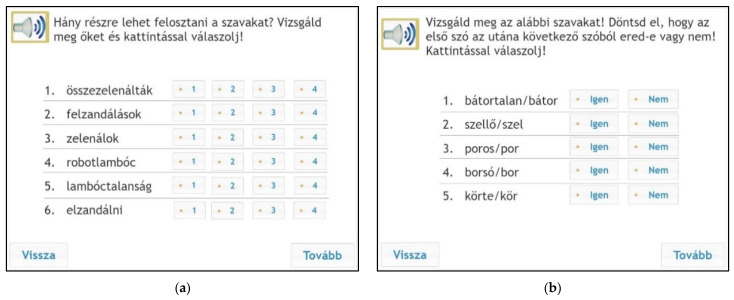
Samples from the morpheme segmentation subtest. Left (**a**): nonwords, instruction: “How many parts do the following words consist of? Answer by clicking”. Options: they have zelened together, 2. upzandalments, 3. I am zelening, 4. robotlambóc, 5. lambóclessness, 6.to zandal away. Right (**b**): relational task, instruction: “Examine the following words carefully. Decide whether we form the first word from the second word? Answer by clicking. Options: discouraged/courage, 2. breeze/breed, 3. dusty/dust, 4. pea/pit, 5. pear/pea.

The testing procedure took place in a group setting in schools’ ICT labs using the eDia platform (Csapó and Molnár 2019; Molnár and Csapó 2019). The entire testing procedure was conducted within a 45 min lesson. Children could listen to the instructions during the test, and they had to provide their answers by clicking on the right answer or dragging words into a box on the monitor (see Figure 1, Figure 2 and Figure 3). Instant feedback was given after completing the test, and the teachers could also download the detailed results within the eDia system with a supplement document which helped them interpret the results. Before the test became available, the schools belonging to the eDia system received a brief description of the instrument assessing children’s morphological awareness and reading comprehension skills. The assessment guideline gave details about the aim of the testing and about how to conduct the data collection. It also explained what skills and subskills it measured, why it could be useful for the children and for the teachers, and included sample tasks as well. The schools were asked to provide headphones for the children willing to take part in the survey. In addition to the scores, teachers could also download a detailed description of the test and personalized feedback for each child. It contained written feedback for different achievement categories and a spider web diagram which showed the performance of a given child as well as the average performance of the given grade. We applied all relevant ethical guidelines of educational research including privacy, confidentiality, and data management during the research process. Parental consent was asked for, and anonym assessment identifiers were used for logging into the test platform.

## 3. Results

### 3.1. Reliabilities and Construct Validity of the Instrument

The reliability of the instrument showed mostly good and acceptable results (Table 3). Based on item-total correlation analyses, we had to exclude one item in the morpheme segmentation subtest because it negatively correlated with the total score of the test. The Cronbach’s Alpha index for the Morphological Structure Awareness test on the whole sample was high in all grades (.93, .91, and .90, respectively). The values were also good in the case of the three main dimensions of morphological awareness (inflectional, derivational and compound morphology). The subtests showed similarly good reliabilities, except for the morpheme segmentation subtest. Nevertheless, this subtest was kept for analyses as well because it assessed important dimensions of morphological awareness. The reading comprehension test worked well and produced acceptable reliabilities in all grades (Table 3).

**Figure 4:**
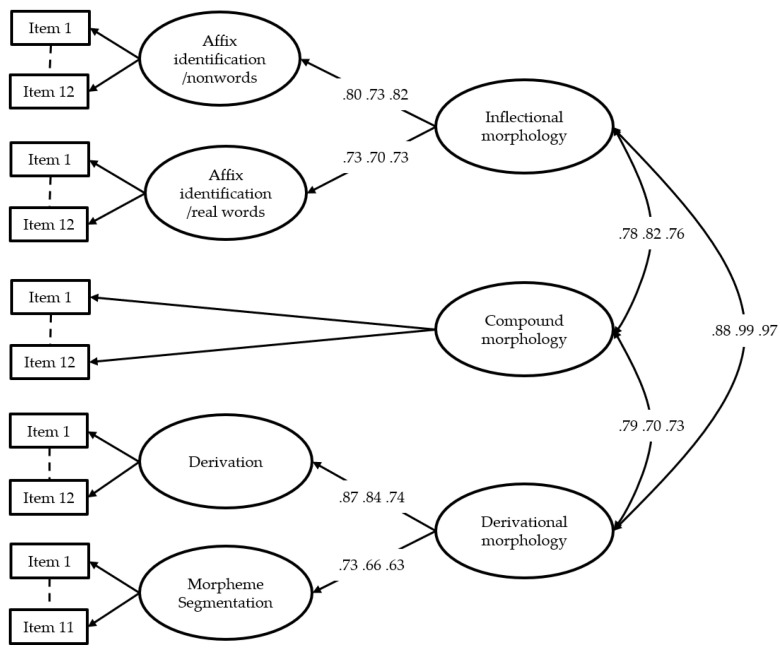
Results of the hierarchical model with factor loadings. The factor loadings are depicted in the order of the grades, respectively (grades 2, 3, and 4).

Confirmatory factor analyses (CFA) were conducted to test the underlying measurement model for morphological awareness. Three different models were tested: a 5-dimensional model based on all subtests; a 3-dimensional model based on the three main dimensions of morphological awareness, namely, inflectional, derivational, and compound morphology; and a 1-dimensional model. Both the 5-dimensional and the 3-dimensinal models showed good or acceptable model fit in all grades; however, according to the chi-squared difference test, the 5-dimensional model fits better to the data compared to the 3-dimensional model in all grades (all grades: χ^2^ = 929.62 *p* < .01; grade 2: χ^2^ = 348.75; *p* < .01; grade 3: χ^2^ = 319.59 *p* < .01; grade 4: χ^2^ = 275.59 *p* < .01). Chi-squared difference tests also showed that both the 5-dimensional and the 3-dimensional model fit significantly better than the 1-dimensional model in all grades (5-dimensional versus 1-dimensional: χ^2^ = 2317.05; *p* < .01; χ^2^ = 960.87; *p* < .01; χ^2^ = 771.56 *p* < .01; χ^2^ = 652.87 *p* < .01, respectively; 3-dimensional versus 1-dimensional: χ^2^ = 1376.62; *p* < .01; χ^2^ = 527.13; *p* < .01; χ^2^ = 444.13 *p* < .01; χ^2^ = 436.96 *p* < .01, respectively). Thus, the five latent factors of morphological structure awareness were empirically distinguished (Table 4). In addition, a hierarchical model was also tested in which inflectional morphology as a further latent factor was determined by the two affix identification subtests, and derivational morphology was influenced by the Derivation and the Morpheme Segmentation subtests. The model also showed good fit indexes in all grades; thus, the three main dimensions of morphological awareness were also empirically confirmed (Table 4). Figure 4 shows the hierarchical model with data parameters in grades 2–4. The factor loadings were high, and the three factors were strongly correlated with each other on a latent level, indicating that the proposed model was consistent with the theoretical assumptions.

### 3.2. Relationships among Morphological Awareness and Its Subtests

Table 5 shows the correlations between the results of the morphological awareness test and all its subtests and between the three main dimensions in all the three grades examined. All subtests are strongly correlated with the morphological awareness test; the coefficients ranged between 0.60 to 0.83. The three main dimensions also strongly correlated with the whole test results, providing further support for construct validity. The magnitudes of correlations between the subtests ranged between .26–.55, indicating that all dimensions represent important and distinguishable aspects of morphological awareness.

### 3.3. The Development of Morphological Skills and Reading Comprehension in Grades 2–4

**Figure 5:**
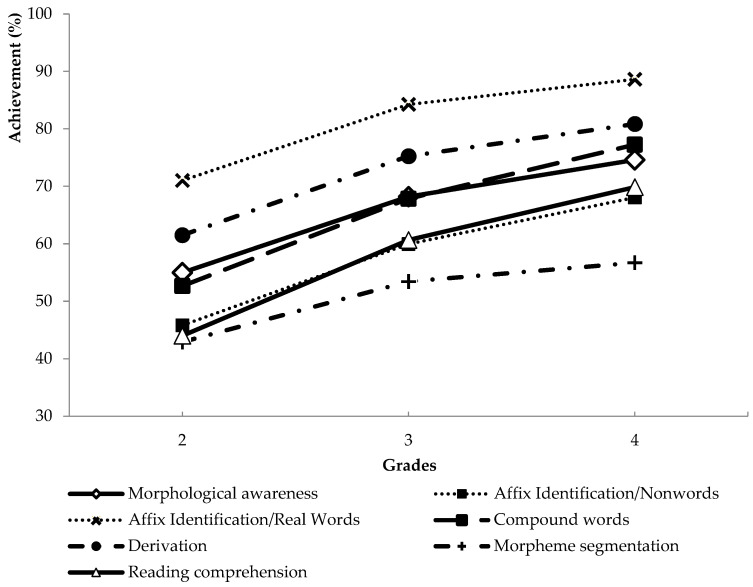
The development of different skills related to morphological awareness and reading comprehension in grades 2–4.

The performance in the test results showed an increasing tendency in morphological awareness (Table 6 and Figure 5). Children’s morphological awareness skills developed throughout the three grades. An analysis of variance (ANOVA) on morphological awareness scores yielded a significant effect of grades, [F (2, 4131) = 433.13, *p* = 0.01]. The post hoc tests showed that the achievements in each grade were significantly different in case of all subtests and dimensions (*p* < 0.05). The easiest tasks were the affix identification for real-words tasks. Compound words, affix identification for real words, and derivation subtests seemed to be harder, and the morpheme-segmentation tasks were the most difficult. Reading-comprehension tasks were rather difficult for the second graders; they became easier for the children in the third grade and fourth grade.

Figure 5 shows the developmental patterns of morphological awareness skills and reading comprehension graphically. Continuous lines represent morphological awareness and reading comprehension. Dashed lines refer to the different subskills of morphological awareness. The same patterns represent the subskills corresponding to a specific dimension (e.g., dotted lines correspond to inflectional morphology). The different skills develop in parallel with each other and with reading comprehension as well. In all cases, a tendency can be noticed that the degree of the development tends to be faster between grades 2 and 3 compared to grades 3 and 4.

### 3.4. The Relationship between Morphological Awareness and Reading Comprehension

Morphological awareness and all the subtests show moderate positive correlations with reading comprehension throughout grades 2–4 (Table 7). An increasing tendency could be observed in the relation of the whole test, but the further examination reveals that inflectional morphology, particularly the Affix Identification for Nonwords subtest, is responsible for this phenomenon. For other subtests, the relationships seem to remain stable throughout the three grades.

A further investigation was carried out to examine the relationships on a latent level as well. Thus, with structural equation modeling, the five subtests were regressed on reading comprehension. The amount of explained variances are high in all grades; the values ranged between 63 and 70% (all grades: 70%, grade 2: 63%, grade 3: 63%, and grade 4: 66%), indicating a strong association between morphological awareness and reading comprehension on a latent level as well. Table 8 shows the regression coefficients and their significance in all grades. The contribution of the different subtests to the total explained variance somewhat differs in each grade; however, a clear tendency can be observed: the most significant predictor of reading comprehension is the Affix Identification for Nonwords subtest. All other subtests’ contributions are notably lower, the explained variances ranged between 2 and 19%, and in some cases, the effect is nonsignificant. Morpheme segmentation seemed to be the least significant predictor for reading comprehension in all grades.

## 4. Discussion

### 4.1. The Psychometric Features of the Online Instrument

Our online assessment tool for morphological awareness proved to be reliable and valid in terms of construct validity. However, the morpheme segmentation subtest should be improved in terms of reliability. The segmentation tasks required a conscious level of morphological awareness because children could rely only on their morphological awareness skills. In addition, this subtest included two types of tasks. Furthermore, students could even listen to the instructions, and examples were provided; however, it is possible that the instructions were difficult for young children, which resulted in a lower performance and weaker associations with reading comprehension. Thus, the lower Cronbach’s Alpha values might be explained by these reasons.

The construct validity is supported by CFA examinations which revealed that the five subtests were empirically distinguishable dimensions of morphological awareness. In addition, inflectional, derivational, and compound morphology as the three main dimensions of morphological awareness were empirically supported by our data. The morphological test seemed to show the difficulties children had in identifying different language elements. The performances in different grades distinguished between children’s performances quite efficiently; therefore, it is assumed that all the subtests of the instrument measured children’s morphological skills efficiently. Significant moderate correlations among the subtests prove that the subtests are integral parts of the construct; thus, they also demonstrate construct validity. As morphological awareness skills play an important role in the development of linguistic intelligence (Gardner 2000; Kornhaber 2019), our test also provides meaningful information about students’ levels and development of language skills and linguistic intelligence as well.

### 4.2. Development of Morphological Awareness in Grades 2–4

The performances in the Morphological Structure Awareness test in the subtests and in the three main dimensions improved from grade 2 to grade 4. Morphological awareness skills improved significantly in all the grades examined. The investigations indicated that the scores in grades 2, 3, and 4 were significantly different; thus, our hypothesis was confirmed. We found that the increase in the development of morphological awareness from grade 2 to grade 3 tends to be faster than the growth between grade 3 and 4. Similar findings were published about the growth in grades 1–4 (Nunes et al. 2003). However, performances in all subtests improved for children who had difficulty in identifying morphemes in multimorphemic words.

Gabig and Zaretsky (2013) postulate that, in grades 3–4, children can complete sentences with the correct pseudoword. Other studies found that solving pseudoword tasks is challenging for children even in grade 4 (Guldenoglu et al. 2012). The post hoc tests showed significant differences between grades 3–4 (Nunes et al. 2003), which means that children’s increasing engagement with the written language increases their awareness of its structure.

Our results gave further evidence that morphological awareness develops and works similarly in a language with a shallow orthography as it does in deep orthographies (Verhoeven and Perfetti 2017; Manolitsis et al. 2019). Children in both shallow and deep orthographies rely on linguistic information when they meet an unknown word (Casalis et al. 2015). However, the fact that they perform better in real-words tasks than in pseudowords tasks implies that their conscious morphological awareness is not fully developed by the end of early primary grades. In nonwords tasks, children have difficulty identifying the base word and the inflections even at the end of the fourth grade. Thus, in line with our expectations, our data provided further empirical evidence that inflectional and compound morphology develop earlier than derivational morphology (Mann and Singson 2003).

We found that children are able to reflect the functional aspects of inflectional morphology by the early elementary grades (Kuo and Anderson 2006). Students are able to identify frequent inflections and common suffixes and prefixes in grades 1–2; however, they experience difficulties when processing words with ir-regular inflections (Gabig and Zaretsky 2013). We also found that the third graders knew more derivational suffixes than the second graders (Anglin 1993). Our research shows that the mastery of derivational allomorphs is not complete by lower elementary grades (4th grade) (Anglin 1993; Carlisle 2000; Tyler and Nagy 1989). Finally, this research also applies to spelling abilities as well, since the morphological test calls attention to the spelling of inflected and affixed words. The affix identification for the nonwords subtest containing some assimilation tasks was difficult for children.

### 4.3. The Relationship between Morphological Awareness and Reading Comprehension

Our results showed a harmonized development of different aspects of morphological awareness and reading comprehension. The correlations among the different subskills for measuring morphological awareness showed moderate correlations with the reading comprehension subtest throughout the three grades. This developmental pattern is in line with our expectations that morphological awareness and reading comprehension are interdependent. This finding also supports the validity of the instrument, and it indicates that morphological awareness has an important role in word reading, which is linked to reading comprehension (Kirby et al. 2012). However, there was an increasing tendency in the association, especially in the case of the Affix Identification for Nonwords subtest (Guldenoglu et al. 2012); the relationships remained stable throughout grades 2–4. Thus, our hypothesis for increasing correlations throughout primary school years was only partly supported by our data. Possible reasons for this finding could be the properties of the reading comprehension test, as it mostly focused on information retrieval skills, or it is also possible that this relationship differs in shallow orthographies. Another interesting result is the relatively strong predictive power of the Affix Identification for Nonwords subtest to reading comprehension on the latent level. A possible reason for this phenomenon is that in these tasks, children are not able to rely on the meaning of the words; therefore, they probably must apply their morphological skills in a more conscious manner.

### 4.4. Limitations and Further Research

Although this work provides a contribution to the literature of morphological awareness, it has certain limitations. Regarding our instrument, the reliability of the segmentation subtest must be improved by further careful investigations of the previously mentioned reasons (e.g., reconsidering the task types or the instructions). Another limitation is that we showed that different aspects of morphological awareness could be assessed through online media; nevertheless, this attribute also can be considered as a limitation on the generalization of other findings. More particularly, the stable correlations between different subskills of morphological awareness and reading comprehension as well as the relatively strong contribution of affix identification for nonwords tasks to reading comprehension could also be influenced by a media effect. Further research should be carried out to investigate the validity of the online tasks. Morphological awareness was usually assessed by traditional face-to-face methods. Our instrument mostly contains multiple-choice questions, which were suitable for automated scoring, but many aspects could not be measured that could have been in open-ended tasks. Thus, a comparison study applying well-established, face-to-face methods and our online instrument on the same sample would be a necessary step for further research. Another fruitful research line would be to implement a longitudinal study to test the predictive validity of the test for different academic achievements. Examining the effects of different aspects of general intelligence such as working memory could also be an area worth studying.

Log-file analyses could also be carried out to learn more about the task-solving behavior of the students and to examine further aspects of how particular items worked and what the common incorrect answers were. With these investigations, we could deepen our knowledge on the underlying thinking processes of morphological awareness and linguistic intelligence.

Further research is also needed to explore the relationship among the different aspects of morphological awareness and the various dimensions of reading skills (e.g., interpretation, integration, and critical evaluation of information). Within this research design, the role of affix identification skills for nonwords tasks could also be investigated. In addition, involving more age cohorts in the research or carrying out a longitudinal study could give further insights into the examination of the stable correlations between different aspects of morphological awareness and reading comprehension throughout primary grades. In addition, an intervention applying affix identification skills for nonwords tasks could also be an area which could add more information related to the contribution of nonwords tasks to the development of morphological awareness, reading comprehension, and linguistic intelligence.

As we conducted our research in a language with a shallow orthography, any generalization to languages with deep orthographies is limited. Thus, further research is needed to examine other similarities and differences between the two orthographies. In addition, as every language has a unique nature, further research should also be carried out in other shallow orthographies to explore whether our findings could be generalized in this narrower context. Nevertheless, the conclusion related to the extent of generalizability of our results for the Hungarian language could be partly held. Although our sample was not representative in a strict sense as in the PIRLS or in the PISA surveys, it was nationwide and covered many aspects of the Hungarian educational system. Therefore, our results provide a generalizable evaluation regarding the developmental level of morphological awareness and its relation to reading comprehension in grades 2–4.

### 4.5. Pedagogical Implications

The pedagogical implications of this research suggest that morphological instruction is an important tool to enhance children’s reading skills, as the skills to manipulate the smallest meaningful parts of the words might be useful when children have to understand multimorphemic words. Our study showed that morphological awareness could be assessed efficiently through online media. It is cost-effective and offers automatic scoring, which makes it possible to receive immediate feedback on students’ performances. The teachers could also download detailed, personalized feedback from the online system and see the differences in the performances within and between classes and the different grades. Based on the scores, teachers and students had the opportunity to identify the subskills which were the most challenging for them and which subskills they would like to improve. Thus, due to the advantages of technology-based assessments, the instrument is a useful, easy-to-use tool for teachers to receive detailed information about children’s morphological skills in grades 2–4.

In addition to the practical aspects of the instrument in assessing morphological awareness in lower elementary grades, our findings also highlighted the importance of applying nonwords in both the assessment and development of affix identification and morphological awareness. Our results suggest that using these task types in learning and instruction could effectively contribute to foster reading comprehension and linguistic intelligence.

## Figures and Tables

**Table 1:** The number of participants and their age (years) in different grades.

	All Grades	Age (M, SD)	Grade 2	Age (M, SD)	Grade 3	Age (M, SD)	Grade 4	Age (M, SD)
total	4134	9.51 (1.03)	1310	8.42 (0.54)	1291	9.47 (0.57)	1533	10.48 (0.63)
boys	2026	9.56 (1.01)	637	8.48 (0.55)	629	9.55 (0.56)	760	10.50 (0.59)
girls	1877	9.44 (1.02)	597	8.36 (0.51)	602	9.39 (0.55)	678	10.44 (0.62)

Note: M: Mean, year. SD: Standard Deviation, year.

**Table 2:** Subtests of the morphological awareness test.

Subtest	Number of Items	Morphological Knowledge
Affix Identification for Nonwords	12	Orthographic awareness, identification of nominal inflections, recognitions of inflections for singular and plural nouns, grammaticality of sentences, and subject–verb agreement (Berko 1958; Apel 2014).
Affix Identification for Real Words	12	Recognition of the stem and affixes, identifying inflections (Kirby et al. 2012), and awareness of syntactic functions of morphemes.
Compound Words	12	Identifying real and pseudo compounds (Apel et al. 2013; Carlisle 2000; Nippold and Sun 2008; Tyler and Nagy 1989).
Derivation	12	Identifying the correct suffixes: awareness of how morphemes are constrained by the stem they are attached to (Carlisle 2000; Nippold and Sun 2008; Tyler and Nagy 1989).
Morpheme Segmentation	12	Identifying the number of morphemes: attaching real affixes to nonwords (Apel et al. 2013); relational knowledge: identifying whether one word comes from another word (Apel et al. 2013; Carlisle 2000).

**Table 3:** The reliabilities of the instrument (Cronbach’s Alpha).

Measures	All Grades (N = 4134)	Grade 2 (N = 1310)	Grade 3 (N = 1291)	Grade 4 (N = 1533)
Morphological Structure Awareness (59 items)	.93	.93	.91	.90
Inflectional Morphology (24 items)	.86	.85	.83	.84
Affix Identification/Nonwords (12 items)	.75	.68	.71	.73
Affix Identification/Real Words (12 items)	.88	.87	.86	.86
Compound Morphology–Compound Words (12 items)	.91	.91	.90	.89
Derivational Morphology (23 items)	.80	.82	.76	.74
Derivation (12 items)	.80	.80	.76	.74
Morpheme Segmentation (11 items)	.67	.67	.61	.64
Reading Comprehension (10 items)	.79	.72	.76	.78

**Table 4:** Goodness of fit indexes for testing dimensionality of morphological awareness.

Model	χ^2^	df	p	CFI	TLI	RMSEA (95% CI)
*All Grades*						
5 dimensions	7122.08	1642	.01	.965	.963	.028 (.028–.029)
3 dimensions	12,190.76	1649	.01	.932	.930	.039 (.039–.040)
1 dimension	21,517.74	1652	.01	.872	.868	.054 (.053–.055)
Hierarchical model	9514.80	1647	.01	.949	.947	.034 (.033–.035)
*Grade 2*						
5 dimensions	2815.64	1642	.01	.974	.973	.023 (.022–.025)
3 dimensions	4155.66	1649	.01	.945	.943	.034 (.033–.035)
1 dimension	7418.58	1652	.01	.873	.868	.052 (.050–.053)
Hierarchical model	3529.29	1647	.01	.959	.957	.030 (.028–.031)
*Grade 3*						
5 dimensions	2981.73	1642	.01	.962	.960	.025 (.024–.027)
3 dimensions	4343.16	1649	.01	.924	.921	.036 (.034–.037)
1 dimension	6679.34	1652	.01	.857	.852	.049 (.047–.050)
Hierarchical model	3456.77	1647	.01	.949	.947	.029 (.028–.031)
*Grade 4*						
5 dimensions	3531.09	1642	.01	.948	.946	.027 (.026–.029)
3 dimensions	4950.62	1642	.01	.909	.906	.036 (.035–.037)
1 dimension	7390.15	1652	.01	.842	.837	.048 (.046–.049)
Hierarchical model	4422.48	1647	.01	.924	.921	.033 (.032–.034)

Note: df: degrees of freedom. CFI: Comparative Fit Index. TLI: Tucker–Lewis Index. RMSEA: Root Mean Square Error of Approximation. χ^2^ and df are estimated by WLSMV.

**Table 5:** Correlations among the Morphological Structure Awareness Test and its subtests.

Measures	MSAT Grades: 2—3—4	Inf. M. Grades: 2—3—4	AINW Grades: 2—3—4	AIRW Grades: 2—3—4	CW–CM Grades: 2—3—4	Der. M. Grades: 2—3—4	D Grades: 2—3—4
Inf. M. (24 items)	.86 .87 .87						
AINW (12 items)	.72 .73 .77	.80 .83 .86					
AIRW (12 items)	.73 .69 .67	.88 .81 .81	.42 .34 .39				
CW–CM (12 items)	.83 .80 .78	.56 .54 .53	.47 .43 .46	.48 .46 .42			
Der. Morph. (23 items)	.85 .83 .82	.58 .58 .55	.53 .52 .52	.45 .43.49	.59 .49 .46		
D (12 items)	.79 .74 .78	.56 .57 .49	.52 .51 .45	.44 .42 .36	.55 .44 .39	.89 .84 .79	
MS (11 items)	.65 .60 .62	.40 .37 .39	.37 .34 .38	.32 .27 .26	.45 .35 .34	.81 .79 .80	.45 .33 .26

Note: MSAT: Morphological Structure Awareness Test. Inf. M.: Inflectional morphology. AINW: Affix Identification/Nonwords. AIRW: Affix Identification/Real Words. CW–CM: Compound Words, Compound morphology. Der. Morph.: Derivational morphology. D: Derivation. MS: Morpheme Segmentation. All correlations are significant at the 0.01 level.

**Table 6:** Means and standard deviations in grades 2–4.

Measures	Grade 2 Mean (SD)	Grade 3 Mean (SD)	Grade 4 Mean (SD)	Effect of Grade (F)
Morphological Structure Awareness	54.98 (20.45)	68.20 (17.48)	74.60 (15.85)	433.13 *p* < .01
Inflectional morphology (24 items)	58.41 (21.61)	72.10 (18,70)	78.34 (17.75)	385.39 *p* < .01
Affix Identification/Nonwords	45.80 (22.53)	59.95 (23.19)	68.05 (22.85)	338.31 *p* < .01
Affix Identification/Real Words	71.01 (28.63)	84.25 (22.48)	88.63 (19.65)	206.71 *p* < .01
Compound morphology–Compound Words (12 items)	52.66 (35.22)	67.84 (32.00)	77.26 (28.00)	214.62 *p* < .01
Derivational morphology (23 items)	52.62 (20.95)	64.34 (17.59)	69.31 (16.18)	305.57 *p* < .01
Derivation (12 items)	61.50 (26.38)	75.25 (21.93)	80.85 (19.28)	269.66 *p* < .01
Morpheme Segmentation (11 items)	42.92 (22.61)	52.43 (21.16)	56.71 (21.56)	145.60 *p* < .01
Reading Comprehension (10 items)	44.00 (26.28)	60.65 (26.94)	69.86 (26.19)	342.17 *p* < .01

**Table 7:** Correlations among different aspects of morphological awareness and reading comprehension.

	MSAT Grades: 2—3—4	Inf. M. Grades: 2—3—4	AINW Grades: 2—3—4	AIRW Grades: 2—3—4	CW–CM Grades: 2—3—4	Der. M. Grades: 2—3—4	D Grades: 2—3—4	MS Grades: 2—3—4
RC (10 item)	.60 .61 .64	.54 .57 .61	.54 .56 .59	.39 .37 .40	.49. 44 .46	.50 .52 .49	.49. 48. 42	.34 .33 .34

Note: MSAT: Morphological Structure Awareness Test. Inf. M.: Inflectional morphology. AINW: Affix Identification/Nonwords. AIRW: Affix Identification/Real Words. CW–CM: Compound Words, Compound morphology. Der. M.: Derivational morphology. D: Derivation. MS: Morpheme Segmentation. RC: Reading Comprehension. All correlations are significant at the 0.01 level.

**Table 8:** Regression analyses on different aspects of morphological awareness and reading comprehension on latent level.

Measure	Coeff.	Sig.	Coeff.	Sig.	Coeff.	Sig.	Coeff.	Sig.
	All Grades		Grade 2		Grade 3		Grade 4	
Affix Identification/Nonwords	0.55	<.01	.52	<.01	.49	<.01	.55	<.01
Affix Identification/Real Words	.10	<.01	.05	=.21	.10	=.03	.14	<.01
Compound Words	.12	<.01	.19	<.01	.09	=.06	.10	=.02
Derivation	.11	<.01	.12	=.04	.16	<.01	.08	=.08
Morpheme Segmentation	.07	<.01	.02	=.70	.10	=.02	.07	=.05

Note: Coeff.: Regression coefficients, Sig.: Significance level, *p*-value. Nonsignificant correlations are titled.

## Data Availability

Not applicable.
